# Antibiotic prescribing pattern in paediatric in patients with first time wheezing

**DOI:** 10.1186/1824-7288-37-40

**Published:** 2011-09-05

**Authors:** Soumya Patra, Varinder Singh, Harish K Pemde, Jagdish Chandra

**Affiliations:** 1 Department of Paediatrics, Lady Hardinge Medical College & associated Kalawati Saran Children's Hospital, New Delhi, India; 2 Department of Cardiology, Sri Jayadeva Institute of Cardiovascular Sciences and Research, Bangalore, Karnataka, India

**Keywords:** Antibiotic, acute bronchiolitis, wheezing

## Abstract

**Background:**

Acute wheezers for the first time in life are an important target group for efforts aimed at reducing unnecessary antibiotic use.

**Objective:**

To evaluate the effect of clinical, laboratory and radiological data on the decision to prescribe antibiotics to paediatric patients with first time wheezing as well as to seek criteria that would justify antibiotic use.

**Methods:**

A prospective study was made of 47 previous healthy children admitted to our hospital with first time wheezing in life between October 2008- March 2009. All the patients were treated as per the treating unit's protocol with oxygen, bronchodilators with or without antibiotics. The cases were analyzed after discharge and the characteristics of those treated with antibiotics (n = 23) were compared with those who were not (n = 24) and analyzed statistically to find the predictors for antibiotic usage.

**Results:**

The mean age of the study groups was 5.8 (+/- 5.1) months. Among the clinical and investigational parameters, presence of predominant crackles and abnormalities on radiograph were the major determinants for antibiotic usage. There were no significant differences in final outcome between these groups.

**Conclusion:**

Antibiotic usage in first time wheezers is still quite prevalent. Presence of crackles and radiological abnormalities often prompt the usage of antibiotics in such cases.

## Introduction

Bronchiolitis is a commonly caused viral infection and is the predominant cause of acute wheezing in infants. It is characterized by acute inflammation, oedema, and necrosis of epithelial cells lining small airways, increased mucus production, and bronchospasm. The most common etiology is the respiratory syncytial virus (RSV), with the highest incidence of RSV infection occurring between December and March [1]. Therapies currently in the treatment of Bronchiolitis include bronchodilator, nasal clearance, ribavarine and/or corticosteroids. While bronchiolitis is an important cause of wheezing in young children, it can also occur, albeit less commonly, with bacterial infections as well. Further, the WHO driven national guidelines on clinical diagnosis and management of pneumonia in the developing nations suggest that fewer than five children with tachypnoea beyond a certain rates should be clinically diagnosed and managed as pneumonia. Therefore antibiotics are often used in the management of first time wheezer in our country and the children with wheezing caused by acute bronchiolitis represent an important target group for efforts aimed at reducing unnecessary antibiotic use. The pediatricians would justify the use of antibiotics in bronchiolitis because it is difficult to differentiate from acute pneumonia or because of the risk of secondary bacteremia or pneumonia in the cases with viral bronchiolitis. Some of these concerns are not substantiated as a prospective study showed that none of their patients with bronchiolitis had bacteraemia [2]. Antibiotic may only be necessary when bacterial pneumonia is suspected e.g. high fever, toxicity, leucocytosis and lobar infiltrate. It has been repeatedly shown that inappropriate use of antibiotic changes the course of the disease and promotes the development of resistant organism but very few studies have been done in developing countries in this regard [3]. This study was conducted to evaluate the effect of clinical, laboratory and radiological data on the decision to prescribe antibiotics to paediatric patients with first time wheezing as well as to seek criteria that would justify antibiotic use.

## Subjects and methods

This was a prospective observational study done in the Department of paediatrics of a tertiary Care centre at New Delhi, between October'2008 to March'2009.

### Study Design

It was a prospective observational study.

### Inclusion criteria

All the children below two years of age admitted with first time wheezing in life were our study population.

### Exclusion criteria

If a child had: i) atopic conditions ii) congenital heart disease and/or iii) known immunodeficiency, (s) he was excluded from the study.

### Methodology

**Figure 1:**
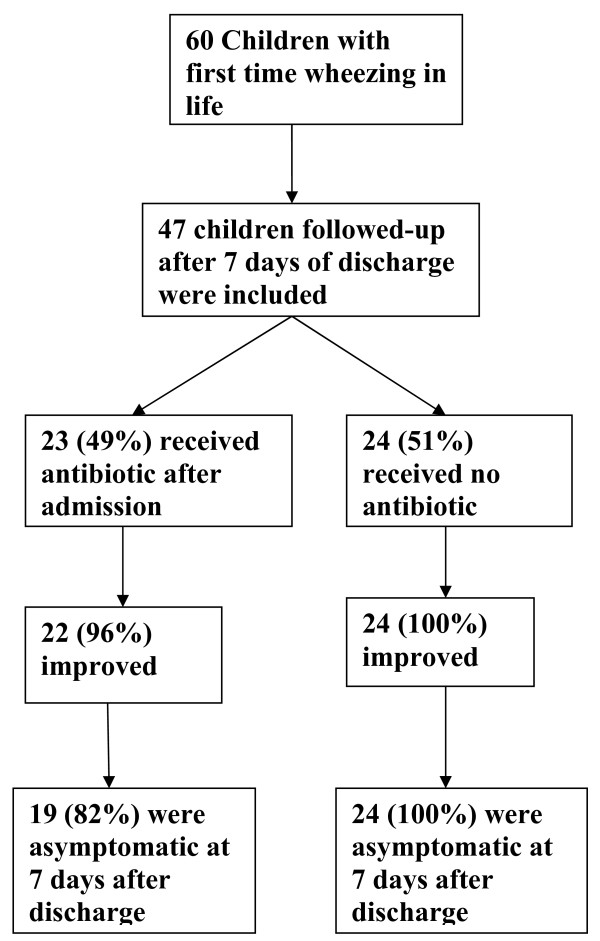
**Study Diagram**.

Structured questionnaire were filled up after taking a detailed history and thorough examination of the child by the investigator. All the patients were subjected to initial investigations such as complete Hemogram, C- reactive protein (CRP), oxygen saturation (SpO2) and/or Arterial blood gases (where necessary) and Frontal view Chest Radiograph. Supportive treatment with Oxygen (SpO2 < 92%) and nebulisation with adrenaline (0.1 ml/kg of 1:1000 solution) or salbutamol solution (0.15 mg/kg) were given at admission and 3-6 hourly after that according to the clinical situation. The cases were followed up three times in 48 hours. The policy of the unit was not universal prescription of antibiotics and the treating/admitting pediatrician could use antibiotics should the clinical or investigative profile suggest the possibility of a bacterial infection (Figure 1). Structured follow- up format included time to resolution of fever, respiratory difficulty and tachypnea, rhonchi and chest indrawing. Criteria for discharging the patient were -feeding adequately, no respiratory distress and no requirement of O2 therapy. The patients were followed-up seven days after discharge in the outpatient department for any recurrence or persistence of symptoms.

The case proformas were evaluated at the end of follow up and the study subjects were divided into two groups- those who received antibiotics (Group 1) and those who did not (Group 2) (Figure 1). The clinical variables between the groups were compared. As the study involved evaluation of the case data after management of the case, no ethical clearance was needed as per Institutional policy. Informed consent of the parents was however taken before enrolling in the study.

### Statistical analysis

Chi-square test was used for statistical analysis using SPSS software and P-value less than < 0.05 was considered as significant result.

## Results

Sixty successive cases with first time wheezer under two years of age were enrolled. Among these, forty-seven children who could be followed up till after seven days of discharge were finally included in this study. Rests were lost to follow-up. Most (70%) of these cases were below six months of age (mean age 5.8 months + 5.1; range 1-24 months). Male children (66%) outweighed the females in number. The prodrome of cough, coryza and fever (85%) along with respiratory distress (96%) was the common presenting feature. Among the presenting signs, all of them had tachypnea and rhonchi in the chest. Among these, 49% (23/47) had received oral or intravenous antibiotics sometime during hospital stay and while about half didn't receive antibiotics. The clinical, laboratory and radiological parameters at admission, course during hospital stay and final outcome of the two groups was compared (Tables 1, 2, 3).

**Table 1:** Comparison between clinical and laboratory parameters at admission

Clinical & Laboratory Parameter	Antibiotic group (n = 23)	Non-antibiotic group (n = 24)	P value/(95% CI)
Mean age	5.3 month (+/- 5.2)	6.3 months (+/- 4.9)	p > 0.1 (1-11)

Viral prodrome	18 (78%)	23 (96%)	P = 0.17 (7-19)

Fever	15 (65%)	09 (38%)	P = 0.1 (-3-10)

Respiratory distress	23 (100%)	23 (96%)	P = 0.9 (21-43)

Tachypnea	23 (100%)	23 (96%)	P = 0.9 (9-17)

Chest indrawing	16 (70%)	12 (50%)	P = 0.28 (2-13)

Rhonchi	23 (100%)	24 (100%)	P = > 0.05 (1-23)

Crepitation	18 (78%)	10 (42%)	p = 0.02 (5-13)

Leucocytosis (> 15,000/cmm)	16 (70%)	11 (46%)	P = 0.18 (14-25)

Abnormal Chest X-ray	16 (70%)	04 (17%)	P = 0.000 (-9-+8)

Increased C-reactive protein (> 1 mg/lit.)	02 (09%)	01 (04%)	P = 0.96 (17-35)

**Table 2:** Comparison of the course during hospital stay between the groups

Clinical parameter	Antibiotic group		Non-antibiotic group		P value
	**After 24 h**	**After 48 h**	**After 24 h**	**After 48 h**	

Fever	08/15	06/15	04/09	00	p > 0.5

Respiratory distress	20/23	05/23	13/23	02/23	P = 0.09

Tachypnea	14/23	07/23	10/23	03/23	p > 0.5

Rhonchi	23/23	21/23	24/24	11/24	P > 0.1

**Table 3:** Comparison between final outcomes within the groups according to their receiving status in patients with first time wheezer

Outcome	Antibiotic group	Antibiotic group	Non-antibiotic
	**(n = 23)**	**(n = 24)**	

Improved	22 (96%)	24 (100%)	P = > 0.5

Deteriorated	01	00	P = > 0.1

Mean Hospital stay	5.86 (+/- 3.2 d)	4.12 (+/- 2.6 d)	p > 0.05

Asymptomatic at follow- up after 7 days	19 (82%)	24 (100%)	P = 0.19

Chest skiagram was done in all cases at admission day and parenchymal opacities like patchy infilterates, segmental atelectasis, and segmental/lobar consolidation were seen in 40% of cases. It was repeated in few cases which did not improve with therapy. Lower mean age at presentation, predominant crackles in the chest and abnormalities on skiagrams like infiltrates, atelectasis, and segmental consolidation were more often associated with antibiotic usage. There was no case with cyanosis or saturation of oxygen < 95%.

The clinical parameters resolved earlier during hospital stay in the Group 2 compared to antibiotic group though the difference between the two groups was not statistically significant.

Almost all patients (96%) of antibiotic treated group and all patients with non-antibiotic group of acute bronchiolitis had improved with treatment. Though the mean hospital stay was more in antibiotic treated group and 18% (4/23) of them had respiratory symptoms at seven days follow-up, but the difference between the two groups was not statistically significant.

## Discussion

In Europe, Australia and North America, about 3% of each year's birth cohort is admitted with bronchiolitis every winter in their infancy [4]. In this study 70% of children were aged below six months and majority of them were male which is in conformity with observations all over the world [5]. Most clinicians assess bronchiolitis on the basis of history and physical examination [1]. Respiratory distress was the most common starting symptoms in our series, though literatures has described fever as being very common in initial phase of illness but it largely disappears by the time of hospitalization as a characteristic feature of the disease [6]. The antibiotic treated group more often had crackles and it appears that the presence of crackles in such cases is often considered as a sign of pneumonia by the clinicians prompting the use of antibiotics. In our study, patients who were sick or had abnormal parenchymal opacities on chest skiagram in the form of infilterates, segmental atelectasis and/or consolidation also were more likely to receive antibioitics. This may not have been necessarily justified because the markers like leucocytosis, increased CRP levels and lobar consolidations often considered suggestive of bacterial infection have also been reported in up to 30% of infants with viral bronchiolitis (most commonly in infants with severe disease) [7]. Many clinicians agree that bacterial pneumonia in infants with bronchiolitis without consolidation is unusual [8] and consolidation by it self does not allow for adequate identification of patients with bacterial infection [9,10]. Consolidation was present in only 7% of our cases but overall parenchymal opacities including patchy infiltration were reported more numerously than others [11,12].

Several studies suggest that the presence of consolidation and atelectasis on a chest radiograph is associated with increased risk for severe disease [13,14] while others found no correlation between chest radiograph findings and baseline severity of disease [15]. Routine radiography in children with bronchiolitis is not recommended because it is nonspecific, fraught with wrong interpretation and adds to unnecessary cost [16,17].

Children with bronchiolitis frequently receive antibacterial therapy because of fever, young age, or the concern over secondary bacterial infection [18,19]. Our study also had similar findings. Mean hospital stay was more in antibiotic treated groups and 18% of them again came with respiratory complaints on seventh day after discharge. This is perhaps a reflection of the practice that sicker and more severely ill children are more often given presumptive antibiotic therapy. Interestingly, the final outcome between the two groups was statistically not significant. Though previous studies showed no benefit from antibacterial treatment of bronchiolitis [18,19] however, the concern remains regarding the possibility of bacterial infections in young infants with wheezing; thus, antibacterial agents still continue to be used.

In our considered opinion, an important message from our findings is that universal usage of antibiotics children with first time wheezing is not needed and efforts should be made to identify test(s) with good discriminatory power to identify those who really need them.

## Conclusion

The problem of antibiotic over usage amongst first time wheezer remains largely due to overlapping clinical presentations, poor laboratory and radiological discrimination and fear of missing bacterial pneumonia in sick babies. Still a significant proportion of the first time wheezers can be managed without any antibiotics. More studies with larger number of subjects are needed to clearly identify those who can be managed without antibiotics.

## Competing interests

The authors declare that they have no competing interests.

## Authors' contributions

VS planned the study, designed the protocol and corrected the manuscript. SP collected data, reviewed the literature and drafted the manuscript. HP and JC were involved in the management of cases. All authors approved the final version of the manuscript.

## Funding

None
